# New Insights on the Evolution of the Sweet Taste Receptor of Primates Adapted to Harsh Environments

**DOI:** 10.3390/ani10122359

**Published:** 2020-12-10

**Authors:** Nur Aida Md Tamrin, Ramlah Zainudin, Yuzine Esa, Halimah Alias, Mohd Noor Mat Isa, Laurence Croft, Mohd Tajuddin Abdullah

**Affiliations:** 1 Department of Animal Science, Faculty of Agriculture, Universiti Putra Malaysia, Serdang 43400 UPM, Selangor, Malaysia; 2 Animal Resource Science and Management Programme, Faculty of Resource Science and Technology, Universiti Malaysia Sarawak, Kota Samarahan 94300, Sarawak, Malaysia; zramlah@unimas.my; 3 Department of Aquaculture, Faculty of Agriculture, Universiti Putra Malaysia, Serdang 43400 UPM, Selangor, Malaysia; yuzine@upm.edu.my; 4 Malaysian Genomics Resource Centre, 27-9 Level 9 Boulevard Signature Office Mid Valley City, Kuala Lumpur 59200, Malaysia; halimah@nibm.my; 5 Malaysia Genome Institute, National Institutes of Biotechnology Malaysia, Ministry of Science, Technology and Innovation, Jalan Bangi, Kajang 43000, Selangor, Malaysia; emno@nibm.my; 6 Centre of Integrative Ecology, School of Life and Environmental Sciences, Deakin University, Geelong 3125, Victoria, Australia; l.croft@deakin.edu.au; 7 Academy of Sciences Malaysia, Level 20, West Wing, MATRADE Tower, Jalan Sultan Haji Ahmad Shah, Kuala Lumpur 50480, Malaysia; abdullahmt@gmail.com; 8 Institute of Tropical Biodiversity and Sustainable Development, Universiti Malaysia Terengganu, Kuala Terengganu 21030, Terengganu, Malaysia

**Keywords:** primate, phylogenetic, sweet taste receptor gene, diet preference, divergence date, late Miocene

## Abstract

**Simple Summary:**

A sense of taste is vital to an animal’s fitness. It enables animals to select and ingest beneficial foods and avoid harmful substances in their daily lives. There have been relatively few studies regarding the evolution of the taste receptor gene for sweetness, particularly in regard to endemic Bornean primates. We constructed *TAS1R2* gene phylogenies for 20 anthropoid primate species using four different methods as well as established the phylogenic time divergence. The phylogenetic analysis successfully separated the primates into their taxonomic groups and as per their dietary preferences. Of note, the estimated time of divergence for the primate speciation pattern in this study was more recent than the previously published estimates. This difference may have been due to environmental changes, such as food scarcity and climate change, during the late Miocene epoch, which likely forced primates to adapt their dietary preferences. These findings establish a foundation for further investigations.

**Abstract:**

Taste perception is an essential function that provides valuable dietary and sensory information, which is crucial for the survival of animals. Studies into the evolution of the sweet taste receptor gene (*TAS1R2*) are scarce, especially for Bornean endemic primates such as *Nasalis larvatus* (proboscis monkey), *Pongo pygmaeus* (Bornean orangutan), and *Hylobates muelleri* (Muller’s Bornean gibbon). Primates are the perfect taxa to study as they are diverse dietary feeders, comprising specialist folivores, frugivores, gummivores, herbivores, and omnivores. We constructed phylogenetic trees of the *TAS1R2* gene for 20 species of anthropoid primates using four different methods (neighbor-joining, maximum parsimony, maximum-likelihood, and Bayesian) and also established the time divergence of the phylogeny. The phylogeny successfully separated the primates into their taxonomic groups as well as by their dietary preferences. Of note, the reviewed time of divergence estimation for the primate speciation pattern in this study was more recent than the previously published estimates. It is believed that this difference may be due to environmental changes, such as food scarcity and climate change, during the late Miocene epoch, which forced primates to change their dietary preferences. These findings provide a starting point for further investigation.

## 1. Introduction

Taste is a vital component of an animal’s fitness. It facilitates animals in making decisions about the ingestion of beneficial foods and avoiding harmful substances in their daily life. Taste evolved to ensure animals choose the appropriate foods for their survival. Taste sensations can be divided into five basic elements: sweet, umami, bitter, salty, and sour [1,2]. Bitter and sour tastes usually warn against hazardous substances, whereas sweet and umami tastes indicate foods that are rich in carbohydrates and proteins, respectively [3]. Salty tastes indicate foods that are rich in sodium and other minerals that are required by animals for optimum biological function.

Sour and salty tastes are conferred by taste receptor cells, which use ion channels, whereas sweet, umami, and bitter tastes are conferred by G protein-coupled receptors (GPCRs) [2,3]. GPCRs comprise two families: taste 1 receptors (TAS1Rs) and taste 2 receptors (TAS2Rs), which function as sweet or umami and bitter taste receptors, respectively [4]. The *TAS1R* gene has a smaller gene repertoire compared with the *TAS2R* gene and comprises three genes in most mammals, namely, *TAS1R1*, *TAS1R2*, and *TAS1R3* [5]. These receptors only function as heterodimers, with *TAS1R3* an obligate partner of both the umami and sweet receptors. The dimer *TAS1R1* + *TAS1R3* acts as the main receptor for umami, whereas *TAS1R2* + *TAS1R3* respond to a variety of natural and artificial sweet ligands [6]. Thus, we can conclude that *TAS1R2* is probably the sole sweet-specific taste receptor gene.

The evolution of *TAS1R* has been less well-studied and characterized compared with the evolution of *TAS2R* because the sweet and umami taste genes are believed to be conserved among species as these genes are necessary for nutrient uptake [7]. However, recently, several studies have demonstrated that the *TAS1R* genes undergo major mutations, revealing new insights regarding the evolution and function of the gene. For example, *TAS1R1* is pseudogenized in the giant panda [8], *TAS1R2* is inactivated in the cat family Felidae [9], and *TAS1R2* is pseudogenized in vampire bats [5]. Consequently, the *TAS1R* gene repertoire is not conserved as predicted. To better understand the evolutionary dynamics of the *TAS1R2* gene, examining species that are closely related to humans that have diverse dietary preferences is necessary.

To obtain a deeper understanding of the phylogenetic relationship between primates’ sweet taste perception, a study of the molecular phylogenetics of the *TAS1R2* gene in primates was conducted. We chose primates because their diets are hugely diverse and include shoots, leaves, plants, fruits, gums, nectars, insects, invertebrates, small mammals, and amphibians (Table 1) [10,11,12,13,14,15]. For simplicity, we classified primates into four main dietary groups, namely, frugivores, folivores, omnivores, and gummivores, to discuss the possible environmental factors and diet preferences that have influenced the evolution of *TAS1R2*.

Recently, a study discovered the oldest fossil of anthropoid primates found in South East Asia, dated ca. 40 million years ago (mya), in the Late Middle Eocene Pondaung Formation (LMEPF), central Myanmar [16]. The LMEPF is internationally famous for bearing terrestrial mammal fossils, including those of anthropoid primates, such as Eosimiidae, Amphipithecidae, Afrotarsiidae, and also extinct early primates (adapiform), and has thus gained much scientific attention [17,18]. The LMEPF provides significant evidence on the colonization, diversification, and origin of anthropoid primates, especially in Asia.

Studies of primates’ divergence dates are controversial and create continuous debates among scientists. Various studies have been conducted to resolve the time divergence of primates using molecular approaches and fossil calibration [19,20,21,22]. The divergence of anthropoid primates (monkeys, apes, and humans) was estimated at 36–50 mya from 54 nuclear gene regions, with 40 mya used as a calibration for the node [20]. The estimated age of divergence, 42.9 mya, determined by a previous study [22] is in line with the date proposed (37.3–52.4 mya). An analysis of mitochondrial genomes placed this node at 46 mya [19], which is earlier than the date proposed by previous authors. Other studies, which used mitochondrial DNA sequences, arrived at estimates of 35 mya [23] and 37.5 mya [24]. These estimated dates are earlier than some of those previously proposed. Sometimes, molecular and paleontological studies disagree, often resulting in the estimated age of divergence for extant primates predating those from the fossil record [25].

Since controversy remains on the divergence time of primates, we will attempt to evaluate their evolution by looking at the traits, such as taste receptors, that are directly linked to the available environmental characteristics (habitats, foods, and forest types) that change through time. This study aimed to construct phylogenetic trees of the *TAS1R2* gene in anthropoid primates to determine the evolutionary dynamics of diet preferences and trace the evolutionary pathway of the sweet taste receptor (time divergence) among species of anthropoid primates.

## 2. Materials and Methods

### 2.1. Ethics Statement and Humane Care Guidelines

Sample collection at Peninsular Malaysia was permitted by the Research Committee of Department of Wildlife and National Parks (DWNP), Cheras, Kuala Lumpur, Malaysia and was approved at Sarawak by the Forest Department of Sarawak under two research permits: NCCD.907.4.4(Jld.IV)-80 and Park Permit No. 37/2011. All sampling procedures and methods performed in this study complied with the rules, regulations, and ethical standards in the treatment of the animals as established by all the relevant wildlife authorities in Malaysia. Furthermore, the guidelines instituted by the Institutional Animal Care and Use Committee, University of California, Davis, USA (as adopted by the PREDICT Project (No. 16,048) Program in Malaysia and under a collaboration between DWNP, Malaysia and Eco-Health Alliance, New York, NY, USA) were followed. All procedures were reviewed and permitted in accordance with Malaysian national laws.

### 2.2. Taxonomic Sampling

Sampling sites were chosen based on the distributions of primate species in Malaysia to collect fresh blood from legal specimens (wild or captive). Blood samples were preserved in ethylenediaminetetraacetic acid (EDTA), and fresh feces were stored at −80 °C on FTA cards. These materials were deposited at the UNIMAS Zoological Museum of Universiti Malaysia Sarawak. A complete list of individuals’ information and GenBank accession numbers for all specimens examined are presented in Supplementary Table S1.

### 2.3. DNA Extraction, Amplification, and Sequencing

The genomic DNA of primate species was isolated from blood and fecal samples using Qiagen DNA Blood Mini Kits (Qiagen, Hilden, Germany) and Qiagen QIAamp DNA Stool Kits (Qiagen, Hilden, Germany). Based on the alignment and consensus of currently available human and rhesus macaque *TAS1R2* sequences, we designed a pair of primers (E6F: 5′-GTAGCCCTGGATCATGCTGT-3′ and E6R: 5′-GCCTTGGTTTCCTCATCTGTG-3′) to amplify ~816 nucleotides from the sixth exon of the *TAS1R2* gene from primates. Polymerase chain reaction (PCR) was conducted using a GoTaq^®^ Flexi DNA Polymerase Promega PCR Kit in a 25 µL reaction containing genomic DNA (15 ng/µL), 10 mM of each primer, 25 mM of MgCl_2_, 10 mM of deoxynucleotide triphosphates, 5× reaction buffer, and 1.25 U of *Taq* DNA polymerase. PCR was conducted under the following thermal profile: 3 min of initial denaturation at 95 °C, 30 cycles of denaturation at 94 °C for 30 s, annealing at 62 °C for 1 min, extension at 72 °C for 2 min, and a final extension at 72 °C for 8 min. Negative and positive controls were used to check for cross-contamination among various components of the reaction and to check the reproducibility of the primers, enzymes, and PCR parameters. DNA amplification was conducted using BIO-RAD DNAEngine^®^ Peltier, BIO-RAD DNA Engine Tetrad^®^ 2 Peltier, or Biometra^®^ TProfessional thermal cyclers. PCR products were examined on 1% agarose gels.

Positive DNA products were purified by centrifugation using Promega Wizard SV Gel and a Promega PCR clean-up system and sequenced at First Base Co. (Selangor, Malaysia) using an ABI 3730 XL DNA Sequencer with a BigDye^®^ Terminator v3.1 cycle sequencing kit on Applied Biosystems’ highest capacity-based genetic analyzer. Each PCR product was sequenced from both directions.

### 2.4. Evolutionary Analyses

CHROMAS (version 2.6.6) [26] software was used to observe and read nucleotide bases of DNA sequences before further analysis. The multiple alignments of the nucleotide sequences were conducted using the CLUSTAL W program [27] and then checked manually by eye. Phylogenetic analyses were conducted using the molecular evolutionary genetic analysis X (MEGAX) [28] software, whereas the Bayesian method was constructed in MrBayes [29]. For the *TAS1R2* dataset, neighbor-joining (NJ), maximum parsimony (MP), maximum-likelihood (ML), and Bayesian methods were used to infer phylogenies. Out of 56 evolutionary models, Modeltest 3.7 [30] showed that the Hasegawa–Kishino–Yano (HKY) model of substitution, with allowance for gamma (G) distribution of rate variation, best fit the data. This model was therefore used for the ML and Bayesian methods. MP analysis was conducted using heuristic searches, with 10 random additions of taxa and tree-bisection-reconnection (TBR) as the branch swapping algorithm. Pairwise genetic distance matrices between and within species were calculated using the Kimura two-parameter model [31], which was applied in MEGAX [28].

### 2.5. Divergence Time Estimates

We conducted a likelihood ratio test on our *TAS1R2* dataset in MEGAX [28] to test the presence of a molecular clock. The Bayesian approach was used to estimate the timescale of diversification on *TAS1R2* gene DNA sequence data from 20 species of primates using paleontological constraints from the fossil record implemented in BEAST version 1.7.5 software [32]. Sequence data from *Myotis lucifugus* was used as the outgroup. Using the uncorrelated lognormal relaxed-clock model, rates were allowed to vary among branches without a priori assumption of autocorrelation between adjacent branches. We also calculated the time of divergence using a strict clock. Based on the results of Modeltest, we assumed the HKY + G model of DNA substitution with five rate categories. Uniform priors were applied for HKY substitution and gamma shape parameters. We applied the Yule process of speciation as the tree prior and an unweighted pair group method with arithmetic mean tree to construct a starting tree within the group assumed to be monophyletic with respect to the outgroup. To obtain the posterior distribution of the estimated divergence times, six calibration points were applied as normal priors to constrain the age of the following nodes (labeled A–F in Table 2). The calibration points selected were based on fossil dates that have undergone extensive review in previous publications and are supported by a consensus of paleoanthropologists (Table 2). Markov chain Monte Carlo runs for each analysis were conducted for 20–100 million generations to ensure sampling of estimated sample size values. Tracer version 1.5 was used to examine convergence, effective sample sizes, and 95% highest posterior density for all mean divergence dates estimated.

## 3. Results

### 3.1. Evolutionary Analyses

Aligned sequences of 816 bp, representing 88% of the total length of the sixth exon of the *TAS1R2* gene (931 bp), were used for the estimation of genetic distance and phylogenetic reconstruction. Of these 816 bp nucleotides, 599 characters were invariant or conserved (73.4%), and 217 characters showed variable sites (26.6%). From 217 characters, 100 variable characters being parsimony-uninformative sites and the remaining 117 characters of parsimony-informative sites. Including the outgroup, 17 informative characters were located at first-codon positions, 30 characters were located at second–codon positions and 70 characters were located at third-codon positions. Parsimony analysis generated a single most parsimonious tree length of 289 with a consistency index of 0.7541 and a retention index of 0.9442. Both ML and Bayesian trees were constructed based on the HKY + G substitution model. The model was the best-fit evolutionary model that was evaluated by script MrAIC provided by the Akaike information criterion in Modeltest 3.07 [39] with a −1 nL of 2653.2 and a gamma distribution shape parameter of 0.4346. Including the outgroup, the average base frequencies used in the analyses were thymine (T) 16.4%, cytosine (C) 23.7%, adenine (A) 24.4%, and guanine (G) 35.5%, indicating an anti-T bias characteristic. Genetic distance within and among each lineage was calculated by the Kimura two-parameter model [31] according to the grouping assignment in the phylogenetic trees (Table 3).

**Figure 1:**
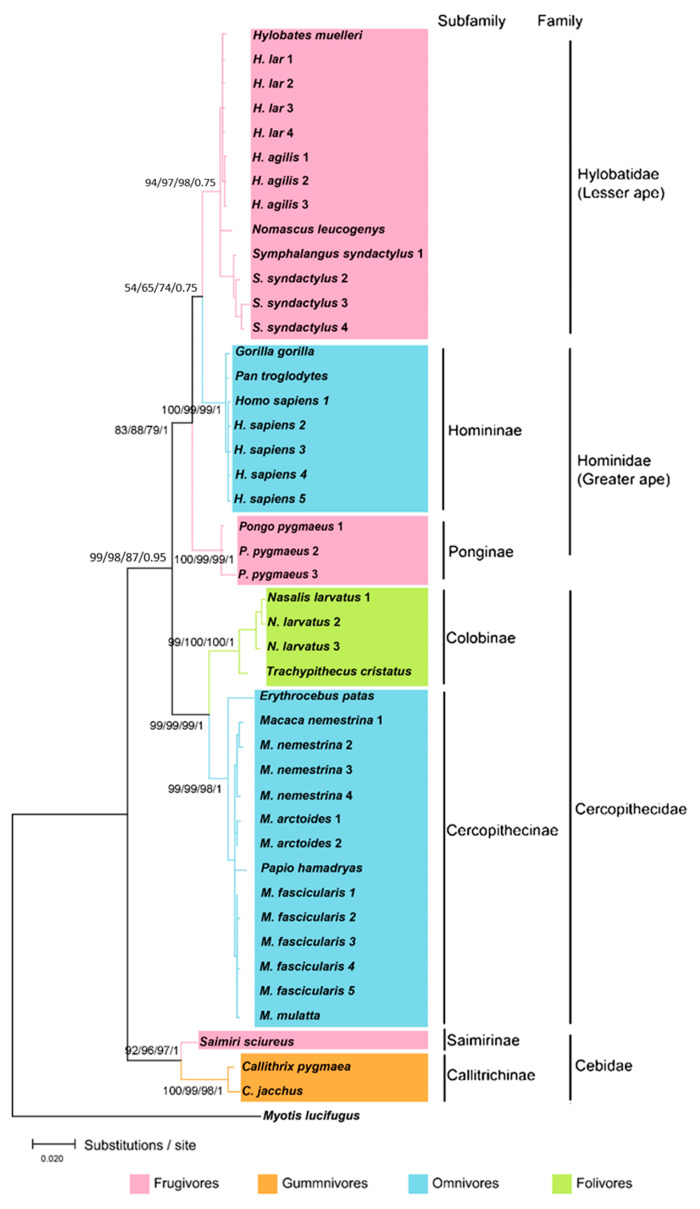
Bayesian phylogram of a 50% majority-rule consensus tree inferred from the alignment of the sweet taste receptor *TAS1R2* gene sequences in exon 6 (816 bp). The phylogram comprises 20 primate species with *M. lucifugus* as an outgroup. Scores on the branches refer to bootstrap support values (1000 iterations) from NJ (1st score), MP (2nd score), ML (3rd score), and Bayesian posterior probabilities (4th score).

From the four methods used to infer the phylogenetic relationships using the *TAS1R2* gene, Bayesian provided the most reliable tree with most of the nodes being supported by high bpp values (Figure 1). According to a previous study [40], Bayesian tree inference tends to outperform the alternative methods because the sequences are evaluated under many models of sequence evolution and often yield estimates that have lower variance than other methods (estimation least affected by sampling errors). They tend to be robust to many violations of the assumptions used in their model [40]. Bayesian inference also includes uncertainty in the probability model, yielding more realistic predictions.

### 3.2. Molecular Clock Analyses

**Figure 2:**
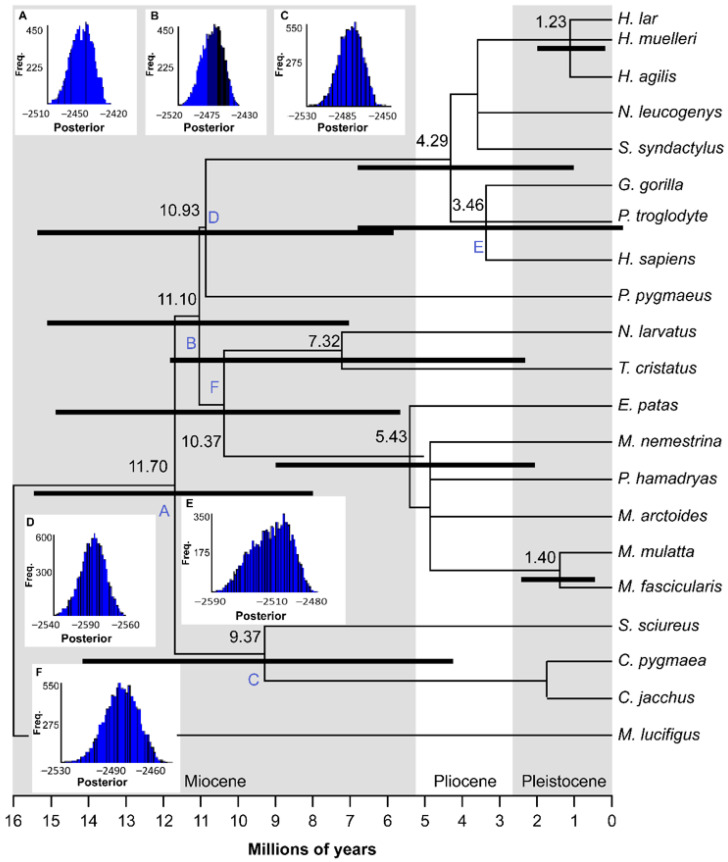
Expanded chronogram of selected single individuals representing each species of primate studied. Values at each node represent estimates of the meantime to the most recent common ancestor from dating analyses performed in BEAST version 1.7.5. Black bars represent the 95% highest posterior density (HPD) intervals for the divergence estimates. Reference fossil dates used for calibration of the tree in dating algorithms are represented by letters A–F on nodes. (**A**) mean = 43.0 MYA, standard deviation (stdev) = 4.5 for time to most recent common ancestor (TMRCA) of Simiiformes. (**B**) mean = 29.0 MYA, stdev = 6.0 for TMRCA of Catarrhini, (**C**) mean = 23.5 MYA, stdev = 3.0 for TMRCA of Platyrrhini, (**D**) mean = 15.5 MYA, stdev = 2.5 for TMRCA of Hominidae, (**E**) mean = 6.5 MYA, stdev = 0.8 for TMRCA of *Homo*-*Pan*, (**F**) mean = 17.6 MYA, stdev = 1.0 for TMRCA of Cercopithecinae and Colobinae. Outgroup (*M*. *lucifugus*) is shown.

The assumption of a strict clock and equal evolutionary rate throughout the tree was significantly rejected by the likelihood ratio test (2 ∆L = 250,628.34), with a 5% significance level (*p* = 0). We provided estimations by using a strict clock (Table 4). However, the expanded chronogram (Figure 2) is based on relaxed-clock analysis only. Our results indicate that the diversification of the primates studied began during the early to middle Miocene. The interspecific diversification dates presented herein should be viewed with caution because, to date, only 20 out of at least 146 species of primate (infraorder Simiiformes) have available *TAS1R2* sequence data. Diversification events between species ranged from 10.93 mya (between *P. pygmaeus* and other apes) to 1.23 mya (between *Hylobates*).

## 4. Discussion

Phylogenetic analyses of partial *TAS1R2* genes in our study resolved six reciprocally monophyletic clades consisting of 20 species of primates from 14 genera, suggesting that old world monkeys (OWMs), new world monkeys (NWMs), and lesser and greater apes formed separate genetic lineage (Figure 1). Furthermore, a small number of polytomies in some nodes highlight the necessity for both genome-scale data and more representative data of each species to resolve interspecific differences.

Some studies have used genetic data to examine the taxonomic statuses and phylogenetic relationships of primates [19,20,33,41,42]. The study that covers the largest number of species is that of [20]. Our study depicted the phylogenetic relationships of living primates with several additional species reviewed, especially those of endangered Bornean endemic species: *N. larvatus*, *H. muelleri*, *P. pygmaeus*, *T. cristatus*, and *C. pygmaea*. We included the species that are native to South America, West and Central Africa and Southeast Asia. The addition of these species is crucial to give more inclusive insights into living primates’ systematic evolution. We included *TAS1R2* sequence data from GenBank in our analyses, thus permitting us to verify the broader interpretation of our clades with specimens from Malaysia and Borneo to the rest of the species of primates available. Below, we discuss the implications of genetic data with respect to their groupings.

### 4.1. NWM (Family Cebidae)

The NWM appears to be the basal lineage in the primate phylogenetic tree in this study. According to our genetic data, the NWM clearly diverged from a common ancestor with OWM and apes roughly 11.70 mya, during the Miocene. Although the direction and nature of primate migration [43,44] and the effect of historic global climate change [45,46] on the NWM remain uncertain, the phylogeny resolves the divergence pattern within the family Cebidae (*Callithrix* and *Saimiri*) and from a common ancestor 9.37 mya.

Our time divergence estimates for NWM are conspicuously more recent than the date proposed by previous studies [20,22], which applied several different molecular datasets using mtDNA and nuDNA. This indicates that the divergence of the sweet taste receptor gene happened more recently than other genes studied. However, the incongruence of divergence dates based on DNA data and fossil calibration can occur for several reasons, including fossil age constraints, tree topology, and models of evolution. The fossil calibration points and tree topology must be cautiously considered because these parameters can generate very dissimilar divergence estimations. Furthermore, a previous study [21] provided optimism that the excavation and reanalysis of fossil materials can also contribute to a reconciliation of the differences in divergence dates between molecular and paleontological studies.

*Saimiri sciureus*, being the basal in NWM in the phylogenetic tree, are larger in body size than the squirrel-sized *Callithrix*; a trend generally similar to findings based on mtDNA [41] and nuclear genes [42]. This grouping is supported by the distinctive diet preferences between *Callithrix* and *Saimiri* and other primates, which feed on gums, sap, and exudates of trees [47,48,49]. This phylogenetic depiction of NWM perhaps reflects the adaptive evolution of their dietary preferences selected by their body size and fluctuating food resource availability within their habitat. The phylogenetic delineation is congruent with several other morphological traits related to dwarfism [50]. There is a case of adaptation termed “phyletic dwarfism,” which is defined as an inclining in morphological size moderately related to evolutionary time [51].

Surprisingly, with evidence of genetic distance, the NWM (especially *S. sciureus*) is more closely related with apes (lesser apes) than with the OWM. This suggests that the *TAS1R2* gene sequences influence the shared diet of frugivores between *S. sciureus* [52] and lesser apes.

### 4.2. OWM (Family Cercopithecidae)

The OWM includes two subfamilies, Colobinae and Cercopithecinae, which were estimated by our genetic data to diverge from a common ancestor with apes 11.10 mya. However, there are differences among genetic data studies, which have different values for time divergence [20,22,36,53]. According to a previous study [20], OWM (family Cercopithecidae) speciation patterns are often bewildered by morphology traits, behavior, and reproduction and are further confused by hybridization between sympatric species and populations.

*N. larvatus* and *T. cristatus* (subfamily Colobinae) split from a common ancestor with subfamily Cercopithecinae approximately 10.37 mya, with species adapted to an arboreal, leaf-eating existence. Although critically endangered *N. larvatus* and nearly threatened *T. cristatus* are OWM, they do not share the same diet as other OWM in the lineage. Both species are specialist folivores and feed on preferably unripe fruits to a lesser extent compared to other OWM [11,54]. For this reason, they are more frequently found in the middle canopy of the forest, leaving the higher branches to fruit-eating primates. They also share unique digestive systems for arboreal folivores primate, having an enormous chambered stomach containing a special cellulose-digesting bacterium that helps to break down the leaves and neutralize the toxins in certain leaves [55]. It is observed and speculated that these species are intolerant toward sweet substances, which can cause them to be bloated if consumed. Both species can be found in the same habitat type of mangrove swamps and other swamp forest regions that are near coastal areas and rivers [10,56]. These habitats provide the same type of food resources. The shared diet, digestive adaptations, and habitat types indicate their close relationship to other OWM, as displayed in the phylogenetic tree.

The OWM lineage follows a pattern of speciation that is characterized by transition from an arboreal to a terrestrial lifestyle and contributes to their type of foraging. The remainder of OWM that arose from a common ancestor, approximately 5.43 mya (99% bootstrap support), are all omnivores, although a few (*M. mulatta* and *M. arctoides*) are primarily frugivores. The species that are omnivorous feed on a variety of foods, including invertebrates, small vertebrates, and plants [15,57,58,59]. The availability of food resources is one of the factors that contribute to how the subfamily Cercopithecinae evolved to become more generalist species, especially the macaques. Macaques exist as commensals to humans and have always been associated with humans by feeding on leftovers and food given by humans and raiding crops and houses, especially long-tailed macaques (*M. fascicularis*), which are considered pests in this country.

### 4.3. Apes and Humans

Apes and humans (superfamily Hominoidea) can be divided into two groups, namely, greater (family Hominidae) and lesser apes (family Hylobatidae). Apes diverged approximately 11.10 mya from the OWM. From the phylogenetic tree, the human sweet taste receptor *TAS1R2* gene is monophyletic with *Pan troglodytes* and *Gorilla gorilla* (100% bootstrap support). These species are omnivorous, but *P. troglodytes* and *G. gorilla* prefer ripe fruits and plants above all other foods [13]. This might be due to the competition and availability of food resources in their habitats, which limits the accessibility of meat.

The highly endangered orangutans comprise the basal group among apes, instead of gibbons as suggested in previous studies [19,20,38]. Inconsistencies are most common when analyzing phylogenetic events in the distant past (e.g., during the Miocene). The phylogenetic incongruence was caused by the more rapid evolution of the *TAS1R2* gene in gibbon lineages compared to orangutan. Such differences may reflect unique selective pressures [60]. Comparing phylogenetic trees estimated from different genes or loci can cause quantitative phylogenetic incongruence through changes in evolutionary rates, horizontal movement of genes and convergent evolution [61].

Ecologically, both orangutans and gibbons favor ripe fruits above all else and eat figs and young leaves as a fallback food in lean periods. However, orangutans are more flexible in their diet and eat more unripe fruit, leaves, bark, and seeds compared to gibbons [62]. Moreover, gibbons are much lighter and travel much faster than orangutans, using their long arms to brachiate at high speed from tree to tree. This enables gibbons to exploit fruit crops on small trees or lianas that are unavailable to orangutans. These dietary preferences and feeding behaviors might be the basis for differences in evolutionary rates of the *TAS1R2* receptor gene between orangutans and gibbons.

The nodes of the apes are not in agreement with most studies using mtDNA and nuDNA [19,20,33], which claimed that families Hominidae and Hylobatidae are sister taxa. Our phylogenetic trees depicted that both families are monophyletic, sharing the same common ancestor and are derived from *P. pygmaeus*. It is assumptive that the evolution of the sweet taste receptor *TAS1R2* gene influenced the apes to be monophyletic. They shared the same diet of fruit eaters, except for humans, gorillas, and chimpanzees, which expanded their diet to be omnivores. These three species are more adaptive and tolerant in terms of their diet.

The genera *Hylobates*, *Symphalangus*, and *Nomascus* (lesser apes) are monophyletic (98% bootstrap value) and rapidly diverged 4.29 mya. From the taxonomic perspective, these three genera are closely related, and this finding is congruent with previous studies [19,20]. From the diet perspective, these genera are mainly frugivorous herbivores (although some may occasionally feed on small amounts of insects) and spend most of their time in the forest canopy searching for food [14,63]. *Hylobates* species appeared more recently than *Symphalangus* and *Nomascus*, with node divergence dates estimated at 1.23 mya. The family Hylobatidae exhibits a period of rapid divergence, perhaps related to gene reorganization, and warrants further research.

### 4.4. Tracing the Evolutionary Pathway: A Reassessment of the Time of Divergence for Anthropoid Primates

Two compelling scenarios were identified and proposed regarding the contradiction in the time of divergence estimates between previous studies and ours.

Scenario one: From the current thinking, the estimated time of divergence for anthropoid primates was ca. 42 mya. Some evidence using genetic datasets and fossil calibration suggested a split for anthropoid primates at 37–47 mya [19,20,22,33,34,35,36,37,38,64,65]. This scenario is further supported by the newly discovered evidence of the oldest fossils of anthropoid primates found in Southeast Asia (Myanmar) aged in the late middle Eocene [16].

Scenario two: Conversely and interestingly, we discovered that the age of divergence for anthropoid primates appeared to be notably more recent than previously proposed, at ca. 12 mya, using the sweet taste receptor gene (*TAS1R2*) dataset with the calibration points chosen based on extensively reviewed fossil dates in other publications, which were supported by paleoanthropologists (Table 2). This scenario suggests that the sweet taste receptor gene diverged and evolved more recently in contrast to other genes studied.

Our estimated age falls in the middle to late Miocene epoch. At this time, the Earth had undergone a series of climatic changes that altered the environmental conditions, especially in the forests. Some of the major climate changes that impacted the environment during the late Miocene to Pliocene are global cooling and the onset of the Asian monsoon, and the El Nino Southern Oscillation [66,67,68]. These occurrences had dramatic effects on temperature fluctuations in the tropics and involved widespread shifts in wind direction that increased the seasonality for rainfall in southern Asia and changes in sea surface temperatures that affect the rainfall patterns across the Pacific [66,68,69,70,71].

Global cooling may have reduced primary productivity and, therefore, food availability in the forests [66]. This cooling, as well as the fall in atmospheric CO_2_ concentration, could have been essential factors behind the retraction of tropical forests and the expansion of grassland during the Miocene. Grassland adapted more efficiently by requiring less CO_2_ for photosynthetic pathways [72,73,74]. This shift in habitat types caused the extinction of many plants, which led to the retreat of tropical forests and the expansion of more seasonal open-canopy woodlands and grassland in the region [75]. Additionally, the temperature dropped by 2–3 °C during the night for a period of 4–8 days during the El Nino years and was the trigger for mast-fruiting events involving primary dipterocarps and many other tree families [66,76,77]. Mast-fruiting events at pre-3–5 mya may not have occurred or at least may not have been of the intensity seen today [66]. This could have been the cause of low food and increased seasonality in fruit availability during the Miocene epoch.

To adapt to the environmental changes and food scarcity of the Miocene epoch while maintaining their survivability, some primates became more generalist feeders, for example, subfamily Cercopithecinae. Some of the greater apes, for example, gorillas and chimpanzees, also become omnivorous from being primarily fruit eaters because larger primates are more affected by these changes than smaller primates [78]. However, some primates, such as the orangutan, proboscis monkey, and silvered leaf monkey, which remained specialist feeders, would surely have contracted their range into less seasonal areas in response to these environmental changes [66]. Eventually, the primates that once inhabited various regions became Bornean endemics. This pattern of changing and maintaining types of feeding can be observed and explained by the evolution of the sweet taste receptor, where it is partly responsible for the feeding behaviors and patterns of primates.

Here, we built on this proposal, with the assessment of time divergence, and evaluated the hypothesis that climate changes that took place during the late Miocene until the Pliocene caused a vast alteration in the environment, forest type, and fruit seasonality, which increased the length of periods of food scarcity (particularly fruits), and that this was the trigger behind the evolution of taste. With the arguments given, we leave the two scenarios for debate.

## 5. Conclusions

This study successfully elucidated the evolution and phylogenetic relationship of the *TAS1R2* gene in primates. Despite the small genetic distance between species, nucleotide variaitons provide sufficient information to separate primates on the basis of their taxonomic groups and dietary preferences. Furthermore, the time of divergence estimation in this study estimates that the geological time of primate speciation occurred more recently than previously published. We believe that this occurred due to environmental and climate change occurrences during the late Miocene epoch. However, further studies focusing on the function of the gene should be conducted to elucidate whether variations in the nucleotides affect the functionality of the gene. Future studies on the evolution of taste should include all other primates with distant relatives such as colugos, flying lemurs, and fruit bats to give a better understanding. The results of this study have greater implications towards conservation for the distinctive *Nasalis larvatus* (proboscis monkey), including its habitats that are facing threats from agricultural development and human settlement projects in coastal regions in Borneo.

## Figures and Tables

**Table 1:** Food preferences of primates found mainly in Southeast Asia and Borneo.

Dietary Groups	Primate	Preferred Diet
Gummivore	Marmosets	Gum, sap, latex, and resin
Folivore	Langurs, snub-nosed monkeys, proboscis monkeys	Shoots, young leaves, and unripe fruits
Omnivore	Macaques, tarsiers, slow lorises, humans, baboons, Patas monkeys, gorillas, chimpanzees	Flexible diets including fruits, leaves, insects, bird eggs, small mammals, and amphibians
Frugivore	Squirrel monkeys, siamangs, gibbons, orangutans	Ripe fleshy fruits and mature leaves

**Table 2:** Fossil dates used as calibration points of divergence time estimates in this study that have undergone extensive review in previous publications and are supported by a consensus of paleoanthropologists.

Divergence (Node)	[33]	[34]	[35]	[20]	[36]	[37]	[19]	[22]	[38]
Crown Anthropoidea (A)	42.8 (40.1–45)	37.7 (33.3–42.7)	45.3 (39.4–51.3)	43.5 (38.6–48.4)	47.2 (38.9–56.5)	40.6 (33.6–49.5)	45.3 (40.7–50.1)	42.9 (37.3–52.4)	46.7 (42.4–50.8)
Crown Catarrhini (B)	29.3 (28–30)	23.9 (23.1–25.9)	30.5 (28.8–35.3)	31.6 (25.7–37.9)	31.0 (25.1–37.7)	25.1 (19.7–32.8)	31.9 (28.3–35.7)	30.5 (26.9–36.4)	32.1 (29.4–33.8)
Crown Platyrrhini (C)	26.6 (23.5–30)	14.5 (9.7–19.9)	N/A	24.8 (20.6–29.3)	25.1 (20.1–31.0)	23.3 (19.2–27.5)	22.0 (19.2–24.4)	20.8 (18.2–24.9)	20.9 (17.9–24.4)
Crown Hominoidea (D)	21.5 (19–24.3)	18.6 (17.1–20.5)	19.9 (16.7–23)	20.3 (16.6–24.2)	19.2 (15.1–24.1)	17.4 (12.4–23.9)	20.3 (17.4–23.5)	18.3 (16.3–20.8)	22.3 (20.5–23.9)
Homo-Pan (E)	N/A	N/A	N/A	6.5 (6.0–7.0)	7.5 (5.7–9.6)	N/A	5.9	6.6 (6.0–7.0)	5.0
Crown Cercopithecinae (F)	23.4 (22–24.9)	13.3 (11.6–14.7)	N/A	17.6 (13.9–21.5)	14.1 (11.0–17.7)	13.2 (8.9–18.3)	22.8 (20.0–25.6)	9.9 (8.9–11.9)	20.8 (18.6–22.9)

Posterior mean and 95% credible interval are in millions of years. N/A, not available. Numbers in the square brackets are the authors cited in references.

**Table 3:** Estimates of evolutionary divergence (%) using the Kimura two-parameter model based on *TAS1R2* gene sequences. Distance values within and among species of primates are shown.

No	Taxon	1	2	3	4	5	6	7	8	9	10	11	12	13	14	15	16	17	18	19	20	21
1	*Hylobates lar*	0.00																				
2	*H. agilis*	0.13	0.00																			
3	*H. muelleri*	0.13	0.26	NA																		
4	*S. syndactylus*	0.93	1.06	1.06	0.00																	
5	*Nomascus leucogenys*	0.66	0.80	0.79	1.34	NA																
6	*H. sapiens*	2.20	2.19	2.35	2.92	2.35	0.00															
7	*P. troglodytes*	2.20	2.19	2.35	2.92	2.65	0.26	NA														
8	*G. gorilla*	2.20	2.19	2.35	2.92	2.65	0.26	0.26	NA													
9	*P. pygmaeus*	2.96	3.10	3.11	3.41	3.43	3.42	3.11	3.43	0.01												
10	*N. larvatus*	6.60	6.76	6.78	7.53	6.79	6.97	7.34	7.34	7.61	0.00											
11	*T. cristatus*	5.48	5.64	5.65	6.37	6.00	5.83	6.18	6.18	6.44	1.39	NA										
12	*M. nemestrina*	5.60	5.75	5.77	6.14	5.78	5.60	5.95	5.95	6.09	4.12	3.43	0.00									
13	*M. fascicularis*	5.60	5.75	5.77	6.14	5.77	5.59	5.95	5.95	6.09	3.79	3.11	0.31	0.00								
14	*M. arctoides*	5.67	5.82	5.85	6.09	5.85	5.67	6.03	6.03	6.08	4.18	3.49	0.28	0.36	0.00							
15	*M. mulatta*	5.56	5.72	5.74	6.10	5.74	5.56	5.92	5.92	6.05	3.76	3.08	0.31	0.03	0.35	NA						
16	*P. hamadryas*	6.07	6.23	6.25	6.62	6.25	5.89	6.25	6.25	6.57	4.55	3.85	0.70	0.69	0.75	0.66	NA					
17	*E. patas*	6.74	6.90	6.93	7.31	6.93	6.74	7.12	7.12	7.26	4.85	4.15	1.81	1.79	1.86	1.76	2.19	NA				
18	*C. pygmaea*	9.05	9.22	9.25	10.37	9.66	10.07	9.65	10.07	10.16	11.50	10.99	10.95	10.96	11.05	10.92	10.96	11.90	NA			
19	*C. jacchus*	9.11	9.28	9.31	10.43	9.31	10.35	9.93	10.35	10.23	11.14	11.06	11.03	11.04	11.13	10.99	11.17	11.98	0.79	NA		
20	*S. sciureus*	7.79	7.96	7.98	8.37	7.98	8.97	8.57	8.97	8.07	10.35	9.45	8.80	8.81	8.89	8.77	8.94	9.69	3.08	3.37	NA	
21	*M. lucifugus*	20.56	20.79	20.86	21.08	20.86	22.36	22.06	22.34	23.37	24.24	23.48	23.88	23.31	24.04	23.31	23.88	25.38	19.97	19.78	20.35	NA

No 1 = white-handed gibbon, 2 = agile gibbon, 3 = Muller’s gibbon, 4 = siamang, 5 = northern white-cheeked gibbon, 6 = human, 7 = chimpanzee, 8 = western gorilla, 9 = Bornean orangutan, 10 = proboscis monkey, 11 = silvered langur, 12 = pig-tailed macaque, 13 = long-tailed macaque, 14 = stump-tailed macaque, 15 = rhesus macaque, 16 = hamadryas baboon, 17 = patas monkey, 18 = pygmy marmoset, 19 = common marmoset, 20 = common squirrel monkey, 21 = little brown bat. NA, not available.

**Table 4:** Divergence times calculated by using relaxed and strict clocks in the expanded chronogram in Figure 2.

Node	Relaxed Clock		Strict Clock	
	Date (mya)	Date (95% HPD)	Date (mya)	Date (95% HPD)
A	11.70	8.27–15.78	10.93	6.89–15.93
B	11.10	7.34–15.40	10.47	6.47–15.66
C	9.37	4.69–14.64	8.84	4.91–13.16
D	10.93	6.59–16.03	11.17	7.21–15.39
E	3.46	0.57–7.82	2.31	0.31–5.16
F	10.37	6.00–15.26	9.75	6.09–13.60

HPD, highest posterior density.

## Supplementary Materials

The following are available online at https://www.mdpi.com/2076-2615/10/12/2359/s1, Supplementary Table S1: Species name, geographic locations, and GenBank accession numbers of *TAS1R2* data used for phylogenetic analyses.
