# Supplementary material for: New Insights on the Evolution of the Sweet Taste Receptor of Primates Adapted to Harsh Environments
Source: Animals (Basel). 2020 Dec 10;10(12):2359. doi: 10.3390/ani10122359 (PMC7764350; doi:10.3390/ani10122359)
Supplement: Supplementary file 1 [file animals-10-02359-s001.pdf]

Supplementary Table S1: Species name, geographic locations, and GenBank accession numbers of *TAS1R2* data used for phylogenetic analyses.

No.	Species name	Field no.	Project no.	GenBank no.	Locality
1	*Callithrix jacchus*	NA	NA	XM002750356	Coastal rainforest, Eastern Brazil
2	*Callithrix pygmaea*	NA	NA	DQ386302	NA
3	*Erythrocebus patas*	NA	NA	DQ386299	NA
4	*Gorilla gorilla*	NA	NA	DQ386296	NA
5	*Homo sapiens* 1	NA	NA	NM_152232.2	NA
6	*Homo sapiens* 2	NA	NA	NM_152232.4	NA
7	*Homo sapiens* 3	NA	NA	BC141437.1	NA
8	*Homo sapiens* 4	NA	NA	AB527225.1	NA
9	*Homo sapiens* 5	NA	NA	BK000151.1	NA
10	*Hylobates agilis* 1	ZM018	PRP0435	KJ806562	Zoo Melaka, Malaysia
11	*Hylobates agilis* 2	ZM001	PRP0055	KJ806564	Zoo Melaka, Malaysia
12	*Hylobates agilis* 3	ZM005	PRP0059	KJ806588	Zoo Melaka, Malaysia
13	*Hylobates lar* 1	ZM006	PRP0060	KJ806580	Zoo Melaka, Malaysia
14	*Hylobates lar* 2	ZM010	PRP0064	KJ806567	Zoo Melaka, Malaysia
15	*Hylobates lar* 3	ZM011	PRP0065	KJ806566	Zoo Melaka, Malaysia
16	*Hylobates lar* 4	ZM020	PRP0063	KJ806561	Zoo Melaka, Malaysia
17	*Hylobates muelleri*	PRP0006	PRP0006	KJ806584	Matang Wildlife Centre, Sarawak, Malaysia
18	*Macaca arctoides* 1	ZM013	PRP0430	KJ806572	Zoo Melaka, Malaysia
19	*Macaca arctoides* 2	ZM014	PRP0431	KJ806576	Zoo Melaka, Malaysia
20	*Macaca fascicularis* 1	PSA002	PRP0067	KJ806569	Cindai, Selangor, Malaysia

21	*Macaca fascicularis* 2	PSA003	PRP0068	KJ806574	Cindai, Selangor, Malaysia
22	*Macaca fascicularis* 3	LKW012	PRP0526	KJ806579	Batu Ara, Langkawi, Malaysia
23	*Macaca fascicularis* 4	PRP0003	PRP0003	KJ806581	Matang Wildlife Centre, Sarawak, Malaysia
24	*Macaca fascicularis* 5	ZM007	PRP0061	KJ806587	Zoo Melaka, Malaysia
25	*Macaca mulatta*	NA	NA	NM_001128089.1	NA
26	*Macaca nemestrina* 1	KG040	PRP0512	KJ806578	Lembah Beriah, Malaysia
27	*Macaca nemestrina* 2	PRP0007	PRP0007	KJ806585	Matang Wildlife Centre, Sarawak, Malaysia
28	*Macaca nemestrina* 3	PRP0012	PRP0012	KJ806563	Matang Wildlife Centre, Sarawak, Malaysia
29	*Macaca nemestrina* 4	PRP0001	PRP0001	KJ806570	Matang Wildlife Centre, Sarawak, Malaysia
30	*Myotis lucifugus*	NA	NA	HM437948	NA
31	*Nasalis larvatus* 1	PRP0005	PRP0005	KJ806573	Bako National Park, Sarawak, Malaysia
32	*Nasalis larvatus* 2	PRP0009	PRP0009	KJ806565	Bako National Park, Sarawak, Malaysia
33	*Nasalis larvatus* 3	PRP0008	PRP0008	KJ806577	Bako National Park, Sarawak, Malaysia
34	*Nomascus leucogenys*	NA	NA	KJ794790	NA
35	*Pan troglodytes*	NA	NA	DQ386295	NA
36	*Papio hamadryas*	NA	NA	DQ386300	NA
37	*Pongo pygmaeus* 1	PRP0010	PRP0010	KJ806571	Matang Wildlife Centre, Sarawak, Malaysia
38	*Pongo pygmaeus* 2	PRP0011	PRP0011	KJ806586	Matang Wildlife Centre, Sarawak, Malaysia
39	*Pongo pygmaeus* 3	NA	NA	DQ386297	NA
40	*Saimiri sciureus*	NA	NA	DQ386301	NA
41	*Symphalangus syndactylus 1*	ZM019	PRP0434	KJ806568	Zoo Melaka, Malaysia
42	*Symphalangus syndactylus 2*	ZM004	PRP0058	KJ806589	Zoo Melaka, Malaysia
43	*Symphalangus syndactylus 3*	ZM008	PRP0062	KJ806575	Zoo Melaka, Malaysia
44	*Symphalangus syndactylus 4*	ZM003	PRP0057	KJ806582	Zoo Melaka, Malaysia

45	*Trachypithecus cristatus*	PRP0004	PRP0004	KJ806583	Bako National Park, Sarawak, Malaysia
